# Impella-Driven High-Risk Percutaneous Coronary Intervention: A Novel, Single Non-Surgical-Centre Case Report

**DOI:** 10.7759/cureus.49128

**Published:** 2023-11-20

**Authors:** Temitope Ajagbe, Olamide Bello, Ona Fagbemi, Tamas Ungvari

**Affiliations:** 1 Cardiology, Royal Cornwall Hospital Treliske, Truro, GBR; 2 Haematology, Somerset NHS Foundation Trust, Taunton, GBR; 3 General Surgery, University Hospital North Midlands Stoke, Stoke-on-Trent, GBR

**Keywords:** high risk percutaneous coronary intervention, case report, rotablation, coronary artery bypass graft, dk crush technique, severely impaired lvef, impella

## Abstract

Complex percutaneous coronary intervention (PCI) procedures have been routinely performed in non-surgical centres in the UK for more than two decades. These procedures follow strict guidelines and recommendations by the British Cardiovascular Intervention Society to ensure a more effective running of PCI programs. Even more so, expected guiding principles necessary for the safe optimisation of complex PCI procedures have also been created.

An 81-year-old male was admitted with non-ST-elevation myocardial infarction (NSTEMI) and severely impaired left ventricle ejection fraction (LVEF; 26% according to the cardiac MRI report). Angiogram findings revealed severe multiple-vessel coronary artery disease affecting the following arteries: right coronary artery (RCA), left anterior descending artery (LAD), left circumflex artery (LCx), and intermediate artery (IM). There was also severe disease in the distal left main stem (LMS) bifurcation extending to the ostia of the LAD, LCx, and IM branches. Following a multidisciplinary meeting, the patient underwent Impella-supported high-risk PCI (complex PCI) using the DK crush technique with no peri- and post-procedure complication and a significant LV function improvement (45-49%). This is the first known case of this procedure performed at the Royal Cornwall Hospital in Treliske (RCHT), Truro, Cornwall.

This case report highlights that when the decision to choose between coronary artery bypass graft (CABG) and PCI is not straightforward following an individualised risk-stratification scoring system analysis and in the setting of patient comorbidities, a high-risk PCI supported with the Impella device is a suitable alternative with promising short-term and long-term outcomes.

## Introduction

There is an increasing need for complex percutaneous coronary interventions (PCI), especially in patients with comorbidities such as frailty and severely impaired left ventricular ejection fraction (LVEF) who have complex coronary lesions and are not unsuitable candidates for revascularisation surgery. Due to the higher potential risk of further reduction in their cardiac output and anticipated recurring episodes of ischemia during the procedure, PCIs were not initially offered to these patients [1,2].

However, revascularisation is now conceivable with PCI thanks to the advent of machine-driven circulatory support devices like the Impella since it sufficiently perfuses the coronaries and systemic circulation [1,2]. Recommendations are now in place to guide the practice of high-risk PCIs, especially in non-surgical centres as there is clear evidence that medical management alone is not sufficient in this group of patients who need high-risk PCIs [3,4]. We report the first successful case of an Impella-driven high-risk PCI at a non-surgical centre in the South of England: the Royal Cornwall Hospital in Treliske (RCHT), Cornwall.

## Case presentation

**Figure 1:**
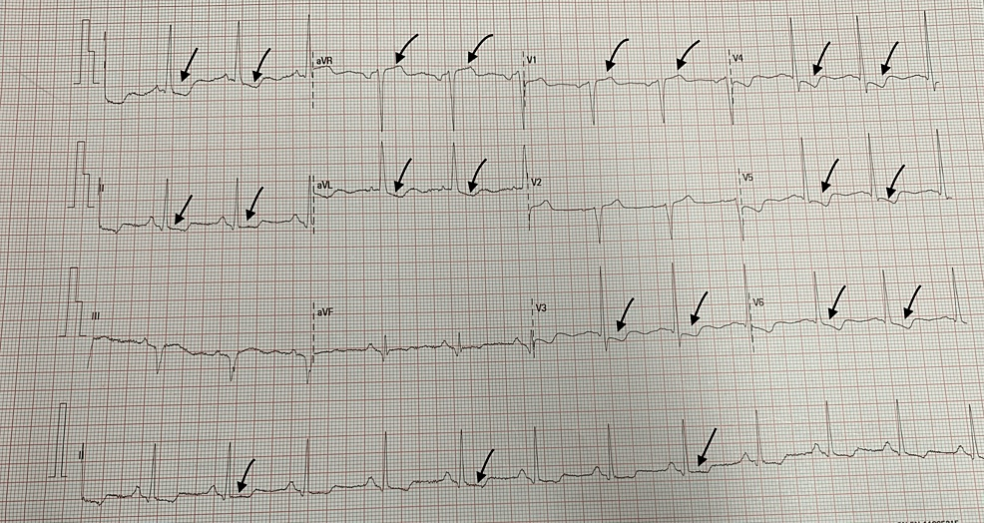
ECG findings on admission ECG: electrocardiogram

An 81-year-old male with a history of hypertension, type 2 diabetes mellitus, asthma, and stage 3 chronic kidney disease was admitted to the Cardiology department following symptoms of chest pain and breathlessness. On admission, he was hemodynamically stable and examination findings revealed normal jugular venous pressure, and heart sounds with no murmurs. He had fine crepitations in both lung bases and bilateral pitting pedal edema. A 12-lead electrocardiogram (ECG), as seen in Figure 1, showed ST depression in I, II, aVL, V3-V6 and mild ST elevation in aVR and V1 suggestive of left main disease. He had elevated troponin on admission (3,100 ng/L; normal value: <14 ng/L) and was diagnosed with non-ST-elevation myocardial infarction (NSTEMI) and was started on ramipril, bisoprolol, aspirin, ticagrelor, atorvastatin, glyceryl trinitrate spray, omeprazole, and diuretics.

Initial transthoracic echocardiography (TTE), as seen in Video 1, showed severely impaired left ventricle (LV) systolic function (LVEF; 30-35%) with akinesis of the mid to apical infero-septal and inferior wall. There was mild mitral regurgitation with normal right ventricle (RV) size and systolic function. He was started on dapagliflozin and eplerenone, and ramipril was replaced with sacubitril/valsartan.

**Video 1:** Video showing severely impaired left ventricle systolic function with regional wall motion abnormalities

**Figure 2:**
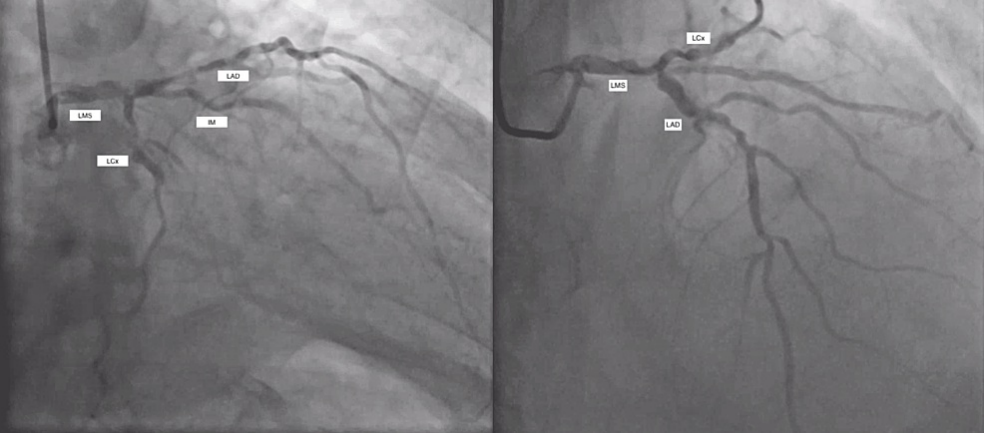
Image showing multi-vessel coronary artery disease LMS: left main stem; LAD: left anterior descending artery; LCx: left circumflex artery; IM: intermediate artery

**Figure 3:**
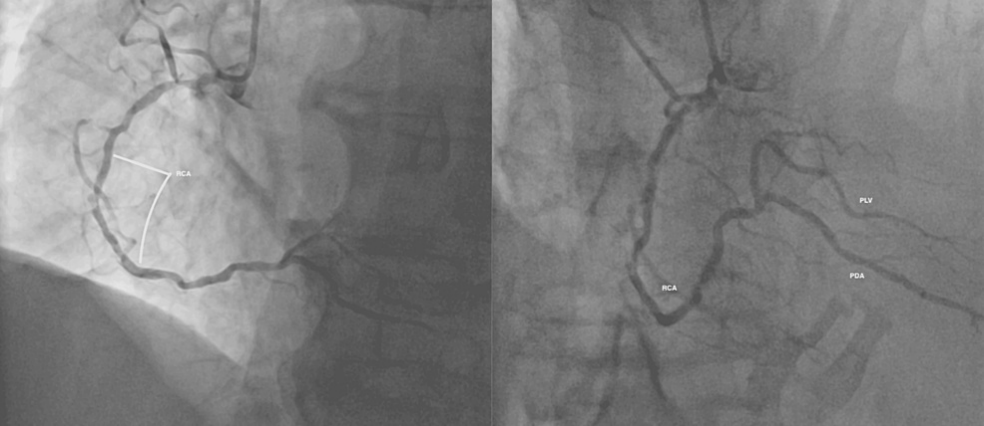
Image showing obstructed RCA with good outflow track to the PLV and PDA branches RCA: right coronary artery; PLV: posterior left ventricular artery; PDA: posterior descending artery

Coronary angiography demonstrated severely calcified multi-vessel coronary artery disease (CAD), as seen in Figure 2 [left main stem (LMS), left anterior descending (LAD), intermediate artery (IM), left circumflex (LCx), right coronary artery (RCA)]. There was severe disease in the distal LMS bifurcation extending to the ostia of LAD, IM, and large LCx branches. In the LCx, the severe disease was diffuse with an occluded first obtuse marginal artery (OM1). The RCA was dominant and also had severe proximal and middle segment disease with a good outflow track as seen in Figure 3.

**Figure 4:**
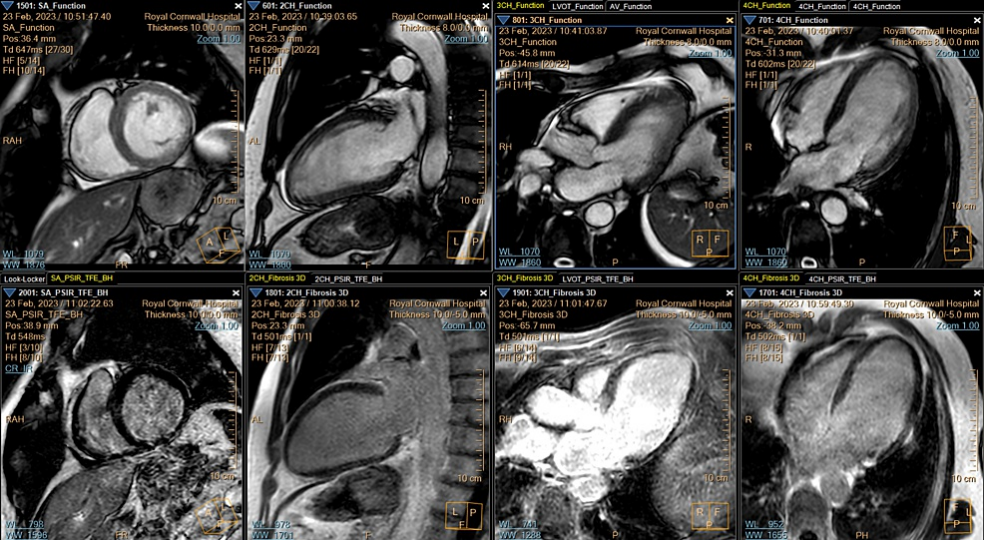
Cardiac MRI images showing viable segments Left to right (top and bottom): cross-section, anterior wall, anteroseptal wall, and septum MRI: magnetic resonance imaging

In view of the patient's TTE findings, a cardiac MRI, as shown in Video 2 and Figure 4, was performed, which revealed dilated, presumably ischaemic cardiomyopathy with an LVEF of 26%. However, no transmural or non-viable infarcted segments were identified.

**Video 2:** Cardiac MRI showing reduced left ventricular ejection fraction with viable segments MRI: magnetic resonance imaging

The patient’s Euroscore II (http://www.euroscore.org) was 4.84%, and his SYNTAX I score (http://syntaxscore.org/calculator/start.htm) was 33. The SYNTAX II scores and four-year mortality rate for PCI vs. coronary artery bypass graft (CABG) are presented in Table 1. 

**Table 1:** SYNTAX II score of the index patient (http://syntaxscore.org/calculator/start.htm) PCI: percutaneous coronary intervention; CABG: coronary artery bypass graft

	PCI	CABG
SYNTAX II score	56	49.5
4-year mortality rate	46.5%	30.4%

The treatment recommendation regarding PCI vs. CABG had to be made by the multidisciplinary team (MDT) involving interventional cardiologists and surgeons and it was agreed that a complex Impella-supported high-risk PCI was the most ideal option given the patient’s risk factors including frailty, advanced age, and multiple comorbidities.

A CT aortogram was done specifically to assess the suitability of the peripheral vessels for large bore access and this showed patent thoracic and abdominal aortas, iliac arteries, and common femoral segments with no evidence of obstructive plaque or any significant tortuosity.

Management and follow-up

An MDT involving interventional radiologists, vascular surgeons, electrophysiologists, and cardiology nurses, including Impella device specialists from the Royal Brompton and Harefield Hospital, and led by interventional cardiologists carried out this procedure under ultrasound (US) guidance and conscious sedation: Impella-supported rotablation and shockwave with PCI to LAD-LCx-LMS (DK crush technique). The 14F Impella CP device was introduced via the right femoral artery using ultrasound guidance following which two perclose were inserted and the Impella was positioned into the LV.

**Figure 5:**
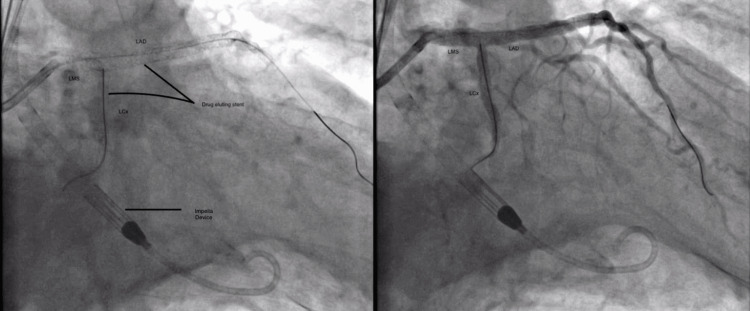
Angiogram images showing the Impella device and drug-eluting stents in the LMS, LAD, and LCx following rotablation LMS: left main stem; LAD: left anterior descending artery; LCx: left circumflex artery

**Figure 6:**
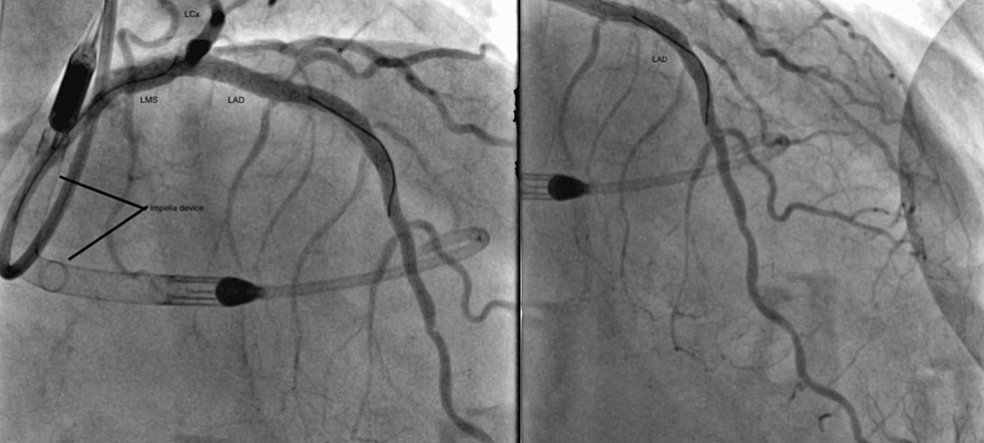
Angiogram images showing good outflow tract through the coronary arteries following drug-eluting stent insertion LMS: left main stem; LAD: left anterior descending artery; LCx: left circumflex artery

After inserting the Impella, the rotablation of the LMS and LAD was done successfully under intravascular ultrasound (IVUS) guidance as seen in Figures 5-6. Two drug-eluting stents (DES) were deployed to the LAD, and one DES each to the proximal LCx and LMS following adequate pre-dilation. The Impella was safely removed and puncture sites were closed with perclose. Immediate post-procedure vascular US and TTE showed no signs of bleeding and effusion respectively. No complications including arterial dissection were noted either intraoperatively or postoperatively.

TTE done 24 hours post-procedure still showed impaired LV function; however, there was a significant improvement in LVEF at 45-49% with only hypokinesia noted in previous akinetic segments, and normal RV size and systolic function with no valvular abnormalities detected.

The patient remained clinically stable and was discharged two days post-procedure and is scheduled for a Cardiology clinic review in three months along with regular assessment of his LV function. Following this, a staged rotablation +/- PCI to RCA would be arranged.

## Discussion

We reported a case of an elderly, frail man with multiple comorbidities admitted due to an NSTEMI and diagnosed with ischemic cardiomyopathy and severely calcified multivessel CAD. This case report illustrates the benefits of Impella for complex PCIs in comparison to medical management only. The Impella device is gaining popularity in high-risk PCIs because it provides adequate sustenance in hemodynamic circulation by improving pressure and providing about 3.5 l/min of cardiac output [5-7]. Although this device has obtained approval in the US and Europe, its use in the UK is currently constrained by factors like the device’s price, limited long-term clinical outcome data, and shortage of experienced staff [8].

The European Cardiology Guidelines recommend risk stratification scoring systems like SYNTAX I, SYNTAX II, and Euroscore to guide the choice of revascularisation surgery and the possibility of PCI in patients who are considered inoperable [1,2]. The SYNTAX scoring system is a grading tool used by specialists to determine the complexity of CAD and it guides in evaluating the prognosis of the disease. A high SYNTAX score correlates with more complex coronary artery disease and overall poor prognosis. This also makes the SYNTAX scoring system a useful tool in predicting the likelihood of a major adverse cardiovascular and cerebrovascular event (MACCE) following revascularisation (PCI or CABG) [9].

**Figure 7:**
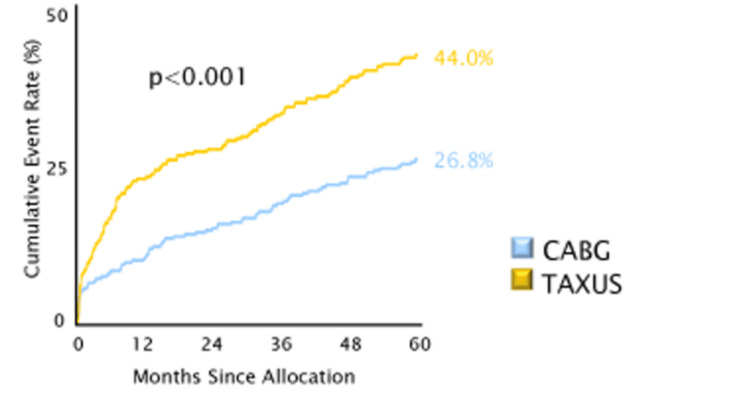
The cumulative MACCE rate in PCI and CABG based on SYNTAX I score MACCE: major adverse cardiovascular and cerebrovascular event; CABG: coronary artery bypass graft

As shown in Figure 7, the chances of MACCE happening are higher following PCI compared with CABG when assessed based on the patient's SYNTAX 1 score. However, this aspect remains to be confirmed in this case as the patient is currently within the first 12 months of follow-up with no complications of post-high-risk PCI so far.

Major reasons as to why the Impella is regarded as a suitable assistive device in high-risk PCI are shown in Table 2 [10].

**Table 2:** Criteria required for a patient to be considered high risk for PCI PCI: percutaneous coronary intervention

In relation to:	Criteria
The patient	Advanced age, heart failure, previous cardiac surgery, presence of valvular heart disease, diabetes, chronic obstructive pulmonary disease, chronic kidney disease, peripheral vascular disease
The hemodynamic state of the patient	High LV end-diastolic pressure, severely impaired cardiac output, anticipated long duration of ischemia, reduced mean arterial pressure, extensive areas of myocardial tissue at risk, ventricular arrhythmias
Anatomy of the coronary arteries	Multiple vessel disease, chronic total occlusion, severely calcified lesion/long lesions, complex lesions at bifurcations, one remaining vessel, unprotected left main vessel

A retrospective study conducted at Queen Elizabeth Hospital, Birmingham, UK has shown similarities in LVEF improvement in 50% of patients when compared to our case, while also recording a low mortality rate (18%) post-Impella-supported PCI [8].

Although RCHT is not regarded as a surgical centre and hence access to the Impella initially presented a challenge, this was resolved by collaborating with the Royal Brompton and Harefield Hospital. Funding is a major limitation in terms of treating more Impella-supported elective cases in non-surgical settings because the UK’s healthcare system is centrally funded as opposed to the insurance-based healthcare models in other nations [8]. However, we hope that more non-surgical centres get funding from the government so that the number of procedures performed can be increased.

## Conclusions

As a bridge to medical therapy alone in enhancing patients' clinical outcomes, we described an Impella-driven high-risk PCI in a non-transplant center in the UK. We strongly hope that this procedure becomes an easily accessible alternative for eligible patients in UK non-surgical settings.
